# Mechanistic Insights into the Ring-Opening Polymerization of Cyclic Esters Catalyzed by Phosphonium Carboxybetaines and Catalyst Design

**DOI:** 10.3390/polym18050663

**Published:** 2026-03-08

**Authors:** Hanghang Li, Wanpeng Xue, Xinyue Zhang, Siyu Ge, Xiaohui Kang, Houli Zhang

**Affiliations:** College of Pharmacy, Dalian Medical University, Dalian 116044, China; lhh1762520517@163.com (H.L.); xuewanpeng22@163.com (W.X.); 15042435922@163.com (X.Z.); gesiyu2024@163.com (S.G.)

**Keywords:** aliphatic polyester, ring-opening polymerization, organocatalyst, phosphonium carboxybetaines, density functional theory

## Abstract

Aliphatic polyesters, widely used in biomedicine due to their biocompatibility and biodegradability, are typically synthesized via the ring-opening polymerization (ROP) of cyclic esters. Although traditional metal catalysts are highly active, their biological toxicity limits their applications. Organocatalysts, particularly natural organic molecules, offer safer alternatives. We explored the ROP mechanisms of cyclic esters (*L*-Lactide (*L*-LA), *ε*-caprolactone (*ε*-CL), and *δ*-valerolactone (*δ*-VL)) catalyzed by phosphonium carboxybetaines (PCBs, (PhR)_3_P^+^(CH_2_)_2_COO^−^, R = H(PCB), F(PCB-F) and OMe(PCB-OMe)) through density functional theory (DFT) computations. The DFT results revealed that the ROP of cyclic esters follows a bifunctional–cooperative activation mechanism, wherein the phosphonium moiety (Ph_3_P^+^(CH_2_)_2_) activates the monomer via an extensive hydrogen-bonding interaction network, and the carboxylate (COO^−^) serves as a proton acceptor to enhance the nucleophilicity of the initiator phenylpropanol (PPA). In contrast, unsubstituted PCB exhibited the lowest energy barrier, being consistent with the highest catalytic activity among PCB derivatives observed experimentally. Moreover, a series of novel PCB derivatives (Ph_3_P^+^(CH_2_)_n_COO^−^, n = 3–6 (PCB1-PCB4)) were designed by regulating the carbon spacer length, and their catalytic performances were computationally tested. The designed catalyst PCB2 (Ph_3_P^+^(CH_2_)_4_COO^−^) exhibited higher activity for the ROP of *L*-LA, attributed to providing sufficient flexibility to minimize deformation while improving proton-accepting capability. Similarly, PCB2 also demonstrated superior catalytic activity for *δ*-VL and the more challenging *ε*-CL monomer. This work not only clarifies the intrinsic catalytic nature of these zwitterionic organocatalysts, but also provides an effective strategy for the rational design of high-performance, metal-free catalysts for the synthesis of sustainable polyesters.

## 1. Introduction

Aliphatic polyesters, as an important class of polymeric materials, are widely used in biomedical applications, including medical devices and drug carriers, owing to their excellent biocompatibility and biodegradability [1,2]. The ring-opening polymerization (ROP) of cyclic esters is an effective approach for the controlled synthesis of aliphatic polyesters with diverse structures and unique properties [3]. In the ROP process, the polymerization efficiency and structure of polymers are largely dependent on the structure and properties of the catalyst. Currently, metal-based catalysts are widely used for their high efficiency and controllability [4,5,6], yet they raise toxicity concerns due to metallic residues, restricting biomedical applications. While less toxic Group IV metal complexes show promise for biocompatible polyesters [7,8,9], they suffer from limited stereoselectivity, poor molecular weight control, and higher costs [10,11,12]. In contrast, metal-free organocatalysts offer selective and diverse activation mechanisms with growing potential in functional polymer synthesis and green chemistry, despite challenges in activity and side-reactions [13,14,15].

In recent years, various natural compounds have been used as catalysts for the ring-opening polymerization (ROP) of cyclic monomers because of their high safety and high catalytic efficiency [16]. Saito et al. used betaine ((CH_3_)_3_N^+^CH_2_COO^−^, chemical name: trimethyl glycine, TMG), a natural compound widely existing in plants such as beets and spinach, which showed excellent catalytic activity for the ring-opening polymerization of cyclic carbonates [17]. Betaine is a zwitterionic organic molecule with a unique structure containing both a quaternary ammonium cation ((CH_3_)_3_N^+^) and a carboxylate anion (COO^−^), endowing it with outstanding catalytic performance in the ring-opening polymerization of cyclic monomers [18,19].

Among betaines, carboxybetaine (R_3_N^+^(CH_2_)_n_COO^−^) is the most abundant [20], while phosphonium carboxybetaine (R_3_P^+^(CH_2_)_n_COO^−^, PCB) is relatively rare. However, phosphorus possesses a larger atomic radius and lower electronegativity than nitrogen, enabling the delocalization of positive charge in phosphonium cations to adjacent groups (such as α-C-H bonds). This enhances the partial positive character of hydrogen atoms, thereby rendering phosphonium cation-based compounds (PCBs) ideal hydrogen-bond donors. In recent years, phosphorus-based organocatalysts have been noted to exhibit high reactivity, excellent stereoselectivity, and tunable structures, enabling superior performance in complex molecule (e.g., pharmaceutical intermediates) synthesis and displaying significant potential in emerging fields such as photocatalysis and electrocatalysis [21,22,23].

Recently, Guo et al. synthesized a series of new phosphonium carboxybetaines ((PhR)_3_P^+^(CH_2_)_2_COO^−^, R = H(PCB), F(PCB-F), and OMe(PCB-OMe)), and used them to catalyze the ROP of *L*-Lactide (*L*-LA), *ε*-caprolactone (*ε*-CL), and *δ*-valerolactone (*δ*-VL) (Table 1) [24]. Among these, unsubstituted PCB achieved the highest turnover frequency (TOF, 46 h^−1^), while PCB-OMe and PCB-F showed slightly lower TOF values (38 h^−1^ and 36 h^−1^). In addition, PCB showed a TOF of only 0.2 h^−1^ for the more challenging *ε*-CL and 0.5 h^−1^ for *δ*-VL. However, the detailed mechanism of PCB-catalyzed *L*-LA polymerization and the internal relationships between activity and PCB structures remain unclear. It is necessary to reveal the mechanism details of polymerization and to further design novel PCB-based catalysts.

Density functional theory (DFT) calculations have been widely used in uncovering the mechanism of the ROP of cyclic esters catalyzed by natural organic compounds. Luo et al. conducted a detailed theoretical study on the ROP mechanism of *L*-LA and trimethylene carbonate (TMC) mediated by DMAP/saccharin (Sac) [25]. In that work, they computationally designed a novel two-component organocatalyst Adenine/Sac with higher activity. Recently, Sardon et al. [16] confirmed that taurine, a naturally occurring amino acid in human and animal tissues, can efficiently catalyze the ROP of *L*-LA and other cyclic monomers under industrially relevant conditions such as 180 °C. Computational studies have shown that its unique zwitterionic structure enables the simultaneous activation of alcohol initiators/chain ends and lactone monomers.

In the present study, we investigated the detailed mechanisms of the ROP of cyclic esters (*L*-LA, *ε*-CL, and *δ*-VL) catalyzed by PCBs using DFT computations. The fundamental reason for the activity difference among PCBs in the polymerization of cyclic esters is elucidated in depth. To further improve the catalytic activity of PCB and provide some valuable insights for experimentation, we computationally designed a series of novel PCBs and predicted their catalytic activity for *L*-LA, *ε*-CL, and *δ*-VL polymerization.

## 2. Computational Details

All calculations were carried out with density functional theory (DFT) using the Gaussian 16 [26] software package. Geometry optimizations, as well as frequency calculations of intermediates (INT) and transition states (TS), were performed with the B3LYP [27] functional and 6-31G(d,p) basis sets at T = 413.15 K. Each optimized structure was subsequently analyzed by harmonic vibration frequencies for the characterization of a minimum (Nimag = 0) or a transition state (Nimag = 1) and to provide thermodynamic data. To further obtain Gibbs free energies (kcal/mol) with a high degree of accuracy, single-point calculations were carried out at the B3LYP-D3BJ [28]/6-311+G(2d,p) level with the SMD [29] solvation model to consider the solvation effect of toluene (ε = 2.37) [30]. To convert Gibbs free energies from 1 atm to 1 mol/L, a correction of *R*Tln(c_s_/c_g_) (about 1.9 kcal/mol) was added to energies of all species. Considering the overestimation of the entropy contribution, a correction of 2.6 kcal/mol was applied to the reaction free energy (i.e., a reaction from *m*- to *n*- components has an additional free energy correction for (*n* − *m*) × 2.6 kcal/mol), based on the free-volume theory [31]. The 3D molecular structures displayed in this paper were drawn using CYLview v1.0 [32]. 

## 3. Results and Discussion

### 3.1. The Mechanism of the ROP of Cyclic Esters Catalyzed by PCB

**Figure 1:**
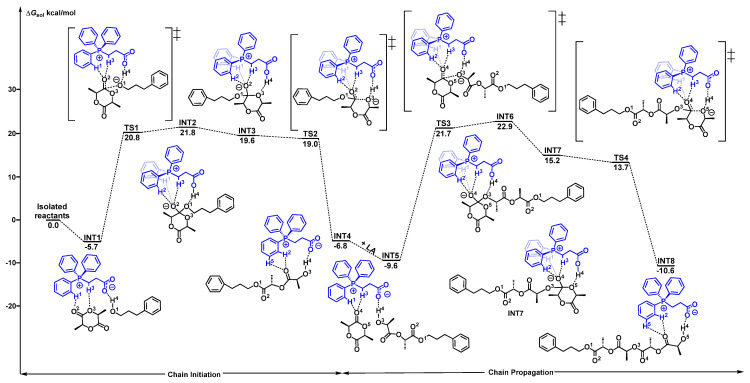
Energy profile of the ROP of *L*-LA catalyzed by PCB (“‡” represents transition state).

Firstly, the polymerization pathway of the *L*-LA monomer catalyzed by the original PCB was calculated. As shown in Figure 1, the catalytic system initially formed a stable ternary complex **INT1** through multiple hydrogen-bonding interactions among the catalyst PCB, the initiator PPA, and monomer *L*-LA. This process released the energy of 5.7 kcal/mol, demonstrating that the formation of the initial complex was thermodynamically favorable.

Furthermore, the carboxylate anion (COO^−^) in PCB, as a proton acceptor, abstracted a H4 atom from the hydroxyl group of PPA. Next, the alkoxide anion performed a nucleophilic attack on the carbonyl carbon of the *L*-LA via **TS1** to form an unstable intermediate **INT2**. This step overcame an energy barrier of 27.5 kcal/mol (21.8−(−5.7) kcal/mol). In the **INT2**, H1 and H2 on the phenyl ring, cooperating with H3 on the α-carbon of the phosphonium side chain, interacted with the carbonyl oxygen of *L*-LA to form triple hydrogen-bond interactions. Subsequently, **INT2** underwent intramolecular hydrogen bond rearrangement to undergo a conformation change to **INT3** by releasing the energy of 2.2 kcal/mol. Finally, the first ring-opened product **INT4** was generated via a barrierless process (**TS2**) with an energy release of 26.4 kcal/mol.

To further understand the polymerization mechanism, the chain propagation process was also calculated. Starting from the ring-opened product **INT4**, the second *L*-LA coordinated with **INT4** through hydrogen bonds to form a stable complex **INT5** with an energy release of 2.8 (−9.6−(−6.8)) kcal/mol. This exoergic process confirmed that the subsequent monomer coordination was thermodynamically favorable. Similar to that of chain initiation, the nucleophilic attack of the new alkoxide anion of **INT5** took place through **TS3** with an energy barrier of 31.3 (21.7−(−9.6)) kcal/mol to generate **INT6**. Subsequently, intramolecular hydrogen bond rearrangement with an energy release of 7.7 kcal/mol and a ring-opening reaction with an energy release of 25.8 kcal/mol of **INT6** occurred sequentially, finally yielding the polymer **INT8**. In the whole process, it was clearly identified that the nucleophilic attack step at the chain propagation stage (with an energy barrier of 31.3 kcal/mol) was the rate-determining step of the polymerization.

**Figure 2:**
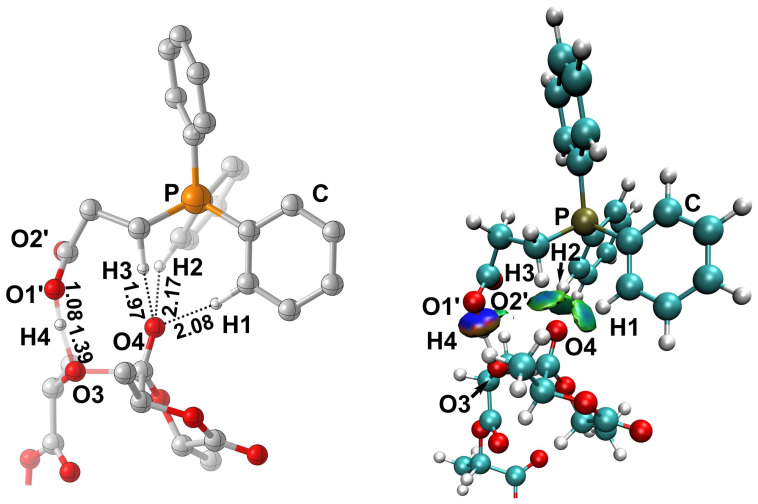
NCI analysis of transition states **TS3** for the ROP of *L*-LA.

It is well known that hydrogen bonding plays a crucial role as a driving force in organic catalytic ROP [33,34,35]. To elucidate in depth the mechanism of the PCB-catalyzed ROP of *L*-LA, the non-covalent interaction (NCI) analysis of the rate-determining **TS3** was carried out. As shown in Figure 2, NCI analysis confirmed that the monomer was activated through the triplet H-binding interactions, as evidenced by the near distances of H1⋯O4 (2.08 Å), H2⋯O4 (2.17 Å), and H3⋯O4 (1.97 Å) between H1 and H2 on the phenyl ring of phosphonium cation and H3 on the α-carbon of the side chain and the carbonyl oxygen O4 of *L*-LA. And the PPA was activated by the carboxylate anion (COO^−^) by abstracting one H4 proton from PPA, as indicated by the distance of O1’⋯H4 (1.08 Å). This bifunctional cooperative mechanism enhances the nucleophilic ability of the alkoxide species to carbonyl carbon of *L*-LA.

**Figure 3:**
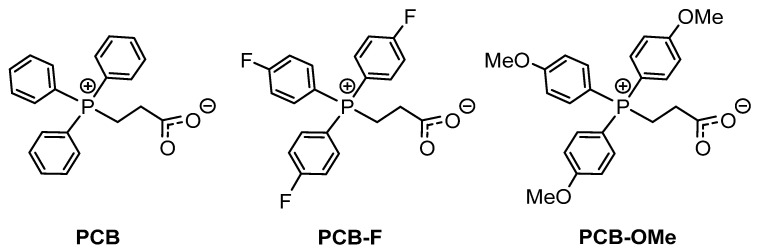
PCBs with different structures.

To explore the substituent effects on catalytic activity, the ring-opening polymerization of *L*-LA mediated by a series of structurally modified phosphonium carboxybetaines with different substituents ((PhR)_3_P^+^(CH_2_)_2_COO^−^), R = H (PCB), F (PCB-F), and OMe (PCB-OMe) were also computationally studied (Figure 3). The calculated results (Table 2) show that PCB catalyzed *L*-LA polymerization exhibited the lowest energy barrier (∆*G*^‡^) of 31.3 kcal/mol of rate-controlling step (**INT5** ⟶ **TS3**) in comparison with the others (PCB-OMe: 32.4 kcal/mol, and PCB-F: 32.9 kcal/mol), showing a strict negative correlation with reported TOF values (PCB (46 h^−1^), PCB-OMe (38 h^−1^), and PCB-F (36 h^−1^)) [24].

**Figure 4:**
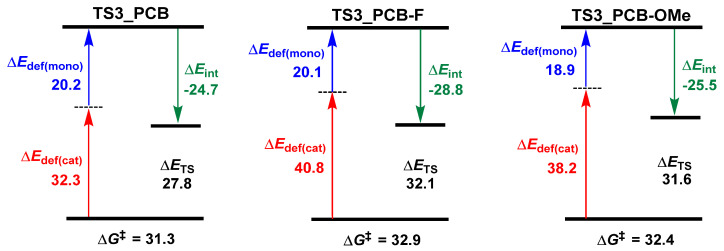
Energy (in kcal/mol) decomposition analyses for **TS3_PCB**, **TS3_PCB-F**, and **TS3_PCB-OMe** (“‡” represents energy barrier).

To further clarify the structure–activity relationship between PCB’s substituents and catalytic performance, energy decomposition analyses (EDAs) for the key transition states (**TS3**) were conducted. As shown in Figure 4, “mono” and “cat” correspond to the LA fragment and the remaining catalyst part in **TS3**, respectively. For PCB, the total deformation energy (Δ*E*_def_) was calculated to be 52.5 kcal/mol (Δ*E*_def(mono)_: 20.2 kcal/mol, Δ*E*_def(cat)_: 32.3 kcal/mol), with an interaction energy (Δ*E*_int_) of −24.7 kcal/mol, resulting in an electronic energy (Δ*E*_TS_ = Δ*E*_def_ + Δ*E*_int_) of 27.8 kcal/mol. In contrast, the PCB-F derivative with electron-withdrawing groups exhibited markedly higher deformation energy (Δ*E*_def_: 60.9 = 20.1 + 40.8 kcal/mol), which made it difficult to offset the stronger interaction energy (Δ*E*_int_: −28.8 kcal/mol), resulting in an elevated Δ*E*_TS_ (32.1 kcal/mol). In comparison with PCB, the PCB-OMe with electron-donating groups also showed increased deformation energy (Δ*E*_def_: 57.1 kcal/mol) and interaction energy (ΔE_int_: −25.5 kcal/mol), leading to a higher Δ*E*_TS_ (31.6 kcal/mol). The excellent correlation between Δ*E*_TS_ values and reaction energy barriers (∆*G*^‡^) validates our computational approach.

**Figure 5:**
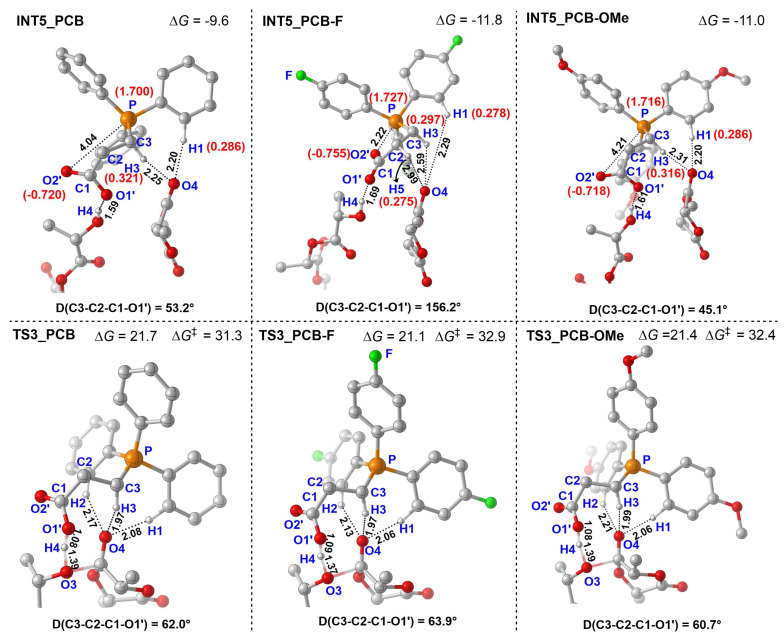
Optimized geometrics of the **INT5** and **TS3** of ROP of *L*-LA catalyzed by PCB, PCB-F, and PCB-OMe (D represents dihedral angle, the NBO charge is shown in red words, energy is in kcal/mol, “‡” represents energy barrier).

To deeply probe the leading role of steric deformation in controlling this activity, the optimized geometrics of **INT5** and **TS3** were comparatively studied (Figure 5). In the fluorine-substituted intermediate **INT5_PCB-F**, the strong electron-withdrawing character of fluorine enhanced the positive charge on phosphorus (natural bond orbital (NBO) analysis: 1.727 vs. 1.700 for **INT5-PCB**). This charge enhancement led to a significantly shorter P⋯O2’ non-covalent distance (2.22 vs. 4.04 Å for **INT5-PCB**) and a stronger interaction between P and O2’. Simultaneously, this effect in fluorine-substituted PCB-F enhanced the delocalization of the phosphonium center (P^+^), increasing the positive charge of H5 (on the distal β-carbon of the carbon chain in **INT5-PCB-F**, (NBO): 0.275 > 0.262 for **INT5-PCB**) and inducing a weak H5⋯O4 hydrogen bond (bond length: 2.99 Å). These changes in interactions—both P⋯O2’ and H5⋯O4 —collectively induced a marked shift in the dihedral geometry of PCB-F. Specifically, the **INT5-PCB-F** complex adopted a highly distorted conformation, with a dihedral angle D(C3-C2-C1-O1’) of 156.2°, compared to that of 53.2° in the unsubstituted systems. This geometric distortion further led to a sharp increase in the dihedral angle change required from **INT5** to **TS3** (92.3°, from 156.2° to 63.9°) in the PCB-F, versus only 8.8° (from 62.0° to 53.2°) for PCB. The change in the dihedral angle explains the significant increase in deformation energy after fluorine substitution on PCB (60.9 vs. 52.5 kcal/mol). For methoxy-substituted PCB-OMe, the electron-donating effect of methoxy weakened P^+^ delocalization, decreasing the NBO charges of H3 (0.316 < 0.321 for PCB) and thus weakening the hydrogen bond (H3⋯O4: 2.31 vs. 2.25 Å for PCB). This reduced the binding constraint between the catalyst and the monomer, thereby enhancing the conformational flexibility of the catalyst molecule and further inducing more pronounced geometric reorganization during the catalytic process, with the dihedral angle change from intermediate to transition state increasing to 15.6° (60.7−45.1°) compared with 8.8° in the PCB system, and the corresponding deformation energy rising to 57.1 kcal/mol versus 52.5 kcal/mol.

In summary, the catalytic activity of PCBs in *L*-LA polymerization is primarily controlled by phenyl ring substituents, where unsubstituted PCB (R = H) shows the lowest energy barrier due to optimal hydrogen bonding and minimal deformation, while electron-withdrawing or donating groups disrupt hydrogen bonds, increase conformational strain, and raise energy barriers, aligning with experimental TOF trends [24].

### 3.2. Catalyst Design

**Figure 6:**
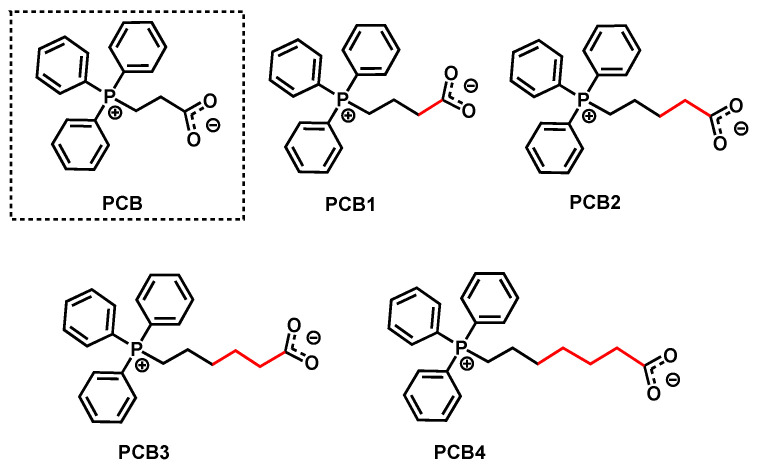
PCB and its designed derivatives.

**Figure 7:**
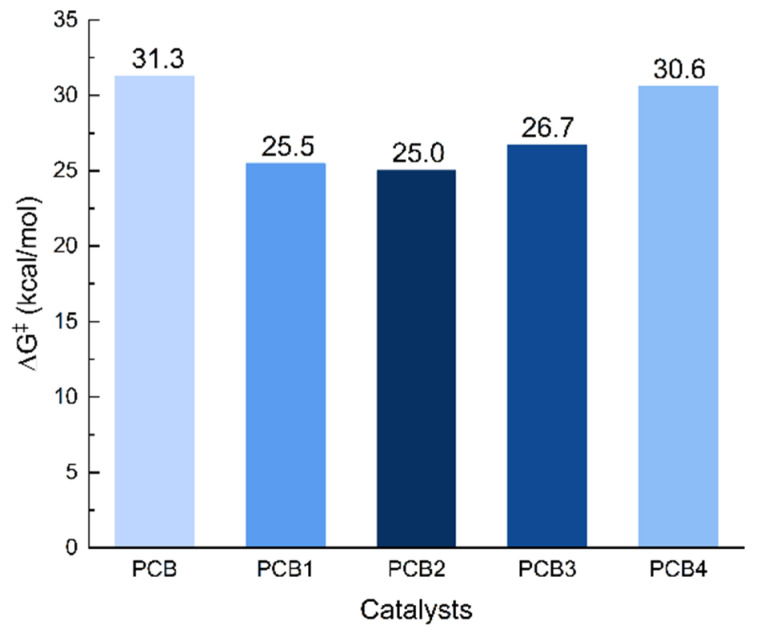
Corresponding energy barriers (∆*G*^‡^) of the ROP of *L*-LA catalyzed by PCB and its designed derivatives (“‡” represents energy barrier).

To enhance the activity of *L*-LA polymerization, we designed a series of PCB derivatives (Ph_3_P^+^(CH_2_)_n_COO^−^, where n = 2 (PCB), 3 (PCB1), 4 (PCB2), 5 (PCB3), and 6 (PCB4) through extending the carbon spacer lengths (Figure 6). This design rationale stemmed from the bifunctional catalytic mechanism of these derivatives, which depends critically on both steric and electronic effects. Optimizing the spacer length involves balancing flexibility—to reduce steric hindrance—and maintaining an intact hydrogen-bonding network that facilitates monomer activation by the phosphonium cation, while simultaneously enhancing proton-accepting capability. In the catalysis of PCB derivatives, the rate-controlling step of *L*-LA ROP was calculated. As shown in Figure 7, it was found that the energy barriers in the catalysis of PCB1, PCB2, PCB3 and PCB4 are 25.5, 25.0, 26.7, and 30.6 kcal/mol, respectively, which are all lower than that (31.3 kcal/mol) of the original PCB case. Therefore, rational adjustment of the spacer carbon chain length can effectively enhance catalytic activity.

**Figure 8:**
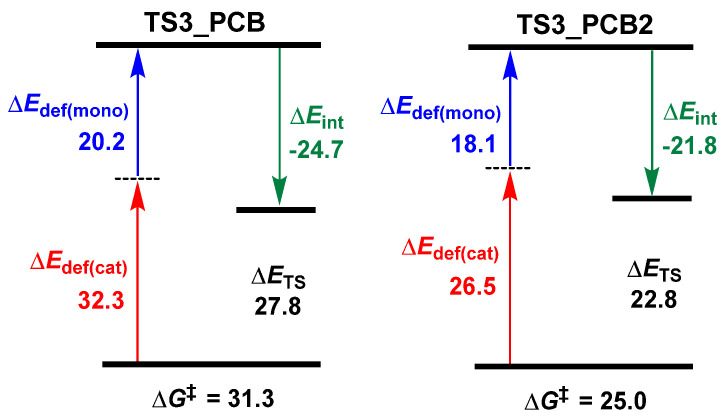
Energy (in kcal/mol) decomposition analyses for **TS3_PCB** and **TS3_PCB2** (“‡” represents energy barrier).

**Figure 9:**
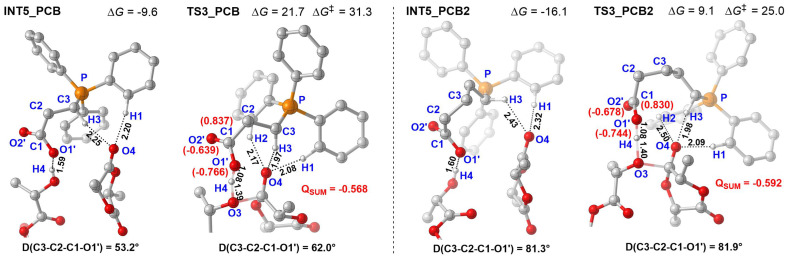
Optimized geometrics of **INT5** and **TS3** of ROP of *L*-LA catalyzed by PCB and PCB2 (D represents dihedral angle, NBO charge is shown in red words, energy is in kcal/mol, “‡” represents energy barrier).

To elucidate the superior catalytic activity of PCB2 over PCB, energy decomposition analysis (EDA; Figure 8) and geometric analysis (Figure 9) were conducted on the key transition states **TS3-PCB** and **TS3-PCB2**. The EDA results reveal that PCB2 exhibits a lower deformation energy (ΔE_def_ = 44.6 kcal/mol vs. 52.5 kcal/mol for PCB) and a stronger interaction energy (ΔE_int_ = −24.7 kcal/mol vs. −21.8 kcal/mol for PCB), collectively resulting in a lower ΔE_TS_ (22.8 kcal/mol vs. 27.8 kcal/mol for PCB). Geometric analysis further indicates a significantly smaller change in the dihedral angle during the INT5 → TS3 transition for PCB2 (0.6°, from 81.9° to 81.3°) compared to PCB (8.8°, from 62.0° to 53.2°), suggesting reduced steric demand that contributes to transition-state stabilization in PCB2. These geometric observations align well with the EDA findings. Further support comes from NBO analysis, which reveals a more negative charge at the anionic center of PCB2 (−0.592) compared to PCB (−0.568). This enhanced charge density improves the proton-accepting capability of PCB2, thereby facilitating the hydrogen transfer process. Therefore, the optimized carbon spacer length in PCB2 affords sufficient flexibility to minimize the structural deformation while enabling effective proton transfer.

Experimental results show that the TOF of *ε*-CL polymerization catalyzed by PCB is lower than that of *L*-LA polymerization. We tested the possibility of *ε*-CL polymerization by PCB and designed PCB2. The energy barrier of the rate-determining step for the ROP of *ε*-CL is 38.8 kcal/mol, which is higher than that of the *L*-LA (31.3 kcal/mol) case. These calculation results show good consistency with the TOF values observed in the experiment (0.2 h^−1^ for *ε*-CL vs. 46 h^−1^ for *L*-LA). The NBO charge differences in the geometric structures of the key intermediates also confirm this observation (Figure S1).

**Figure 10:**
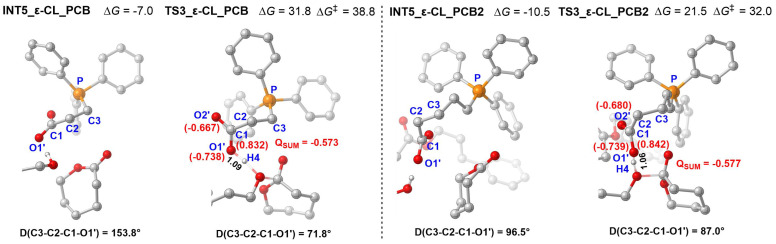
Optimized geometrics of the **INT5** and **TS3** of ROP of ε-CL catalyzed by PCB and PCB2 (D represents dihedral angle, the NBO charge is shown in red words; energy is in kcal/mol, “‡” represents energy barrier).

To improve the polymerization activity of *ε*-CL, the designed PCB2 was also used to catalyze the ROP of *ε*-CL. As shown in Figure 10, it was found that the energy barrier of the *ε*-CL catalyzed by PCB2 was 32.0 (21.5−(−10.5)) kcal/mol, which is lower than that (38.8 kcal/mol) of the PCB case. The same geometric and NBO analyses for **INT5**-PCB(*ε*-CL), **TS3**-PCB(*ε*-CL), **INT5**-PCB2(*ε*-CL), and **TS3**-PCB2(*ε*-CL) were carried out. The more negative NBO charge (−0.577 vs. −0.573 of PCB) of carboxylate anion in **TS3**-PCB2(*ε*-CL) and the smaller dihedral angle rotation (9.5° (96.5−87.0°) vs. 82.0° (153.8−71.8°, in PCB)) effectively reduce the energy barrier of *ε*-CL ROP, enabling the catalyst PCB2 to catalyze the polymerization of *ε*-CL. PCB2 demonstrates high experimental feasibility for *ε*-CL polymerization under practical conditions.

To further verify the superior catalytic activity of PCB2, we calculated the polymerization of *δ*-VL catalyzed by PCB and PCB2. These calculated results show that the energy barrier of the rate-determining step is 34.1 (30.6−(−3.5)) kcal/mol for PCB and 25.9 (16.7−(−9.2)) kcal/mol for PCB2. The energy barrier of *δ*-VL falls between those of *L*-LA and *ε*-CL (34.1 vs. 31.3 and 38.8 kcal/mol for PCB, 25.9 vs. 25.0 and 32.0 kcal/mol for PCB2), which is consistent with the experimental TOF trends. Compared with PCB, PCB2 lowers the energy barrier by 8.2 (34.1−25.9) kcal/mol and thus enhances catalytic activity, further supporting the high experimental feasibility of PCB2.

## 4. Conclusions

In this work, we employed DFT computation to systematically investigate the ROP of cyclic esters (*L*-LA, *ε*-CL, and *δ*-VL) catalyzed by PCBs. The calculation results indicate that the ROP of *L*-LA follows a bifunctional cooperative activation mechanism. That is, the phosphonium cation activates the monomer through a hydrogen-bonding network, while the carboxylate anion enhances the nucleophilicity of the initiator, PPA, by abstracting a proton. This study demonstrates that substituents regulate the catalytic activity by influencing the hydrogen bond interaction network, thereby affecting spatial conformational deformation. Finally, we designed a series of new PCB derivatives by prolonging the carbon spacer length between Ph_3_P^+^ and COO^−^. Among the designed PCB derivatives, PCB2 exhibits the highest activity in comparison with PCB and other PCB derivatives in the ROP of *L*-LA, *δ*-VL and the more challenging *ε*-CL, which was attributed to PCB2’s lower demand for steric adjustment and stronger proton-accepting ability. This theoretical study provides in depth theoretical insights into the polyester synthesis reactions catalyzed by PCBs and the design of novel catalysts based on PCB. Further experimental validation and new catalyst screening based on PCB catalysts will be carried out in future work.

## Figures and Tables

**Table 1:** The polymerization of *L*-LA, *ε*-CL, and *δ*-VL catalyzed by PCB (initiator: phenylpropanol (PPA)) [24].

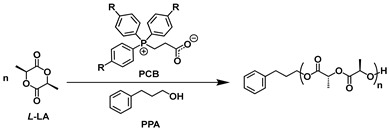					
Monomer	Catalyst	[M]_0_/[PPA]_0_/[Cat]_0_	Time (h)	Conv. (%)	TOF (h^−1^)
*L*-LA ^a^	PCB (R = H)	25:1:1	0.5	92	46
*L*-LA ^a^	PCB-F (R = F)	25:1:1	0.5	76	38
*L*-LA ^a^	PCB-OMe (R = OMe)	25:1:1	0.5	72	36
*ε*-CL ^b^	PCB (R = H)	25:1:1	48	31	0.2
*δ*-VL ^b^	PCB (R = H)	25:1:1	48	96	0.5

a. The temperature for ROP of *L*-LA was 140 °C, in the bulk. b. The temperature for ROP of *ε*-CL and *δ*-VL was 90 °C, in the bulk.

**Table 2:** Energies (kcal/mol) of intermediates and transition states in different PCBs (“‡” represents energy barrier).

PCBs	∆*G*_INT5_	∆*G*_TS3_	∆*G*^‡^
PCB	−9.6	21.7	31.3
PCB-F	−11.8	21.1	32.9
PCB-OMe	−11.0	21.4	32.4

## Data Availability

Original contributions presented in this study are included in the article/Supplementary Material. Further inquiries can be directed to the corresponding authors.

## Supplementary Materials

The following supporting information can be downloaded at: https://www.mdpi.com/article/10.3390/polym18050663/s1, all optimized coordinates are provided in coordinates.xyz file.
