# Supplementary material for: Mechanistic Insights into the Ring-Opening Polymerization of Cyclic Esters Catalyzed by Phosphonium Carboxybetaines and Catalyst Design
Source: Polymers (Basel). 2026 Mar 8;18(5):663. doi: 10.3390/polym18050663 (PMC12987023; doi:10.3390/polym18050663)
Supplement: Supplementary file 1 [file polymers-18-00663-s001.zip › SI.pdf]

Supporting information

Mechanistic Insights into the Ring-Opening Polymerization of Cyclic Esters Catalyzed by Phosphonium Carboxybetaines and Catalyst Design

Hanghang Li [1], Xiaobing Zhang [2], Wangpeng Xue [1], Xinyue Zhang [1], Siyu Ge [1], Xiaohui Kang [1]*, Houli Zhang [1]*

[1] *College of Pharmacy, Dalian Medical University, Dalian, 116044, China*

[2] *The Second Hospital of Dalian Medical University, Dalian, Liaoning, China*

* Correspondence to: kangxh@dmu.edu.cn (Xiaohui. Kang), houlizh@163.com (HouLi. Zhang)

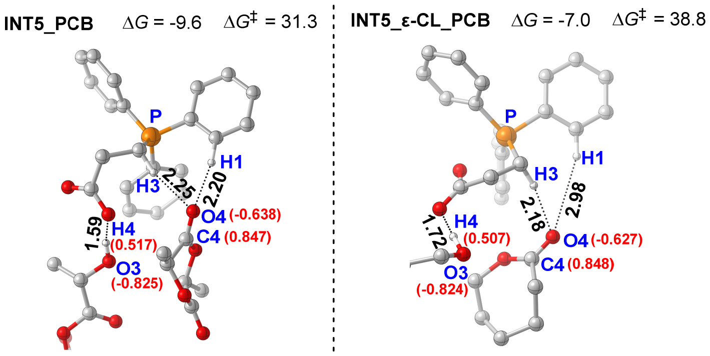

Figure S1. The optimized geometrics of the **INT5** for the ROP of *L*-LA and *ε*-CL catalyzed by PCB (the NBO charge is shown in red font, energy is in kcal/mol, "‡" represents energy barrier).

Table S1. Energies (kcal/mol) of intermediates and transition states in the gas phase.

Structures	ΔG_{gas}
TS1	12.5
INT2	12.6
TS3	11.2
INT6	10.7
